# Supplementary material for: Synergistic Inhibitory Effect of Berberine and Low-Temperature Plasma on Non-Small-Cell Lung Cancer Cells via PI3K-AKT-Driven Signaling Axis
Source: Molecules. 2023 Nov 27;28(23):7797. doi: 10.3390/molecules28237797 (PMC10708101; doi:10.3390/molecules28237797)
Supplement: Supplementary file 1 [file molecules-28-07797-s001.zip › molecules-2651104-supplementary.pdf]

SUPPLEMENTARY MATERIALS

Synergistic Inhibitory Effect of Berberine and Low-Temperature Plasma on Non-Small-Cell Lung Cancer Cells via PI3K-AKT-Driven Signaling Axis

Table of Contents

1. Figure S1. Effects of berberine and/or LTP on cell cycle progression(Flow cytometry)--- -S3

2. Figure S2. Effects of berberine and/or LTP on cell apoptosis (Flow cytometry)-----------S4

3. Table S1. Total RNA purity and integrity determination results-----------------------------S5

4. Table S2. Filtered reads quality statistics--S6

5. Table S3. Statistical table of reference genome comparison results-------------------------S7

6. Figure S3. Correlation between A549 and H1299 cell samples--------------------------------S8

7. Figure S4. PCA analysis results ---S9

8. Figure S5. Distribution of gene expression levels of each sample----------------------------S10

9. Figure S6. Density diagram of expression volume of each sample --------------------------S11

10. Figure S7. Stacking map of gene expression of each sample----------------------------------S12

11. Figure S8. Volcanic map showing gene differences between groups-------------------------S13

12. Figure S9. Protein interaction network diagram--S14

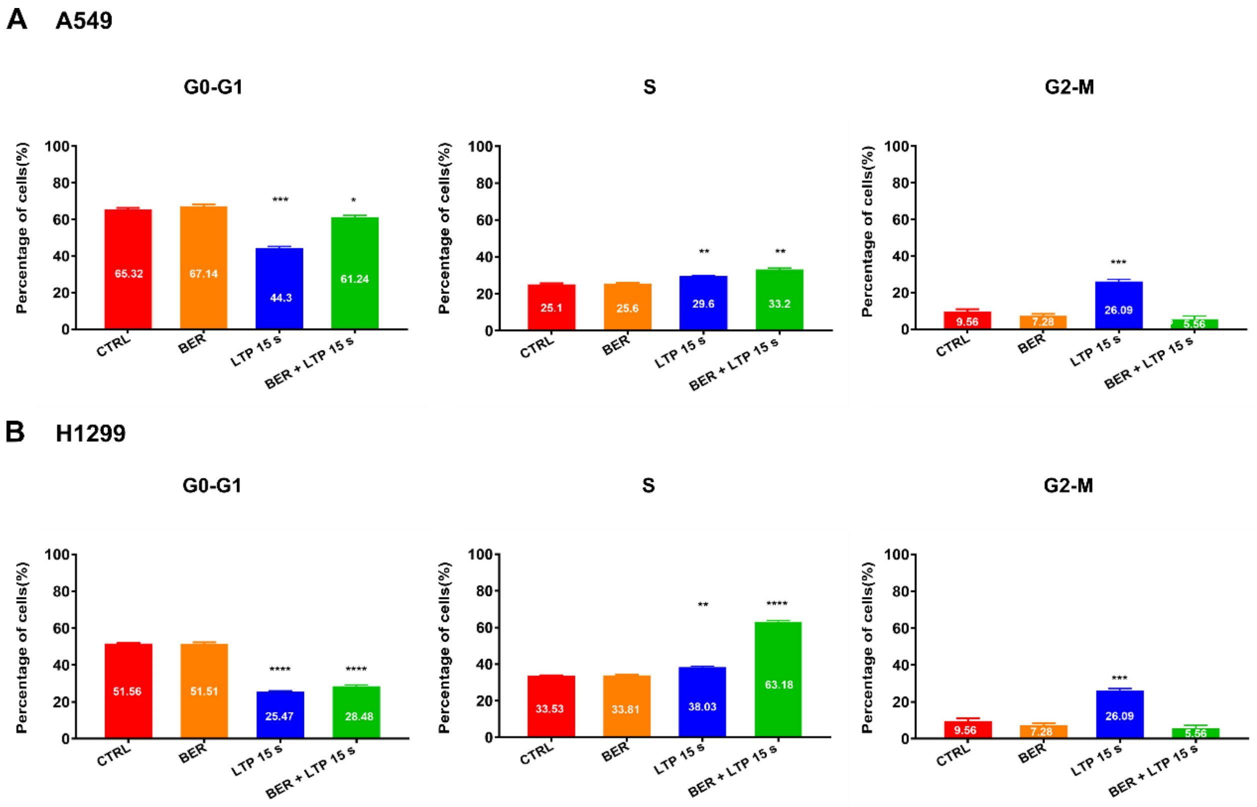

Supplementary Figure S1. Effects of berberine and/or LTP on cell cycle progression (Flow cytometry). (A) BER and/or LTP treatment affected the cell cycle progression on A549 cells. (B) BER and/or LTP treatment affected the cell cycle progression on H1299 cells. Data were presented as the Mean ± SEM of three independent experiments. Statistical significance was calculated using T-test to compare each treatment group to the corresponding vehicle control; significance is indicated by asterisks: *$p < 0.05$, **$p < 0.01$, ***$p < 0.001$, ****$p < 0.0001$.

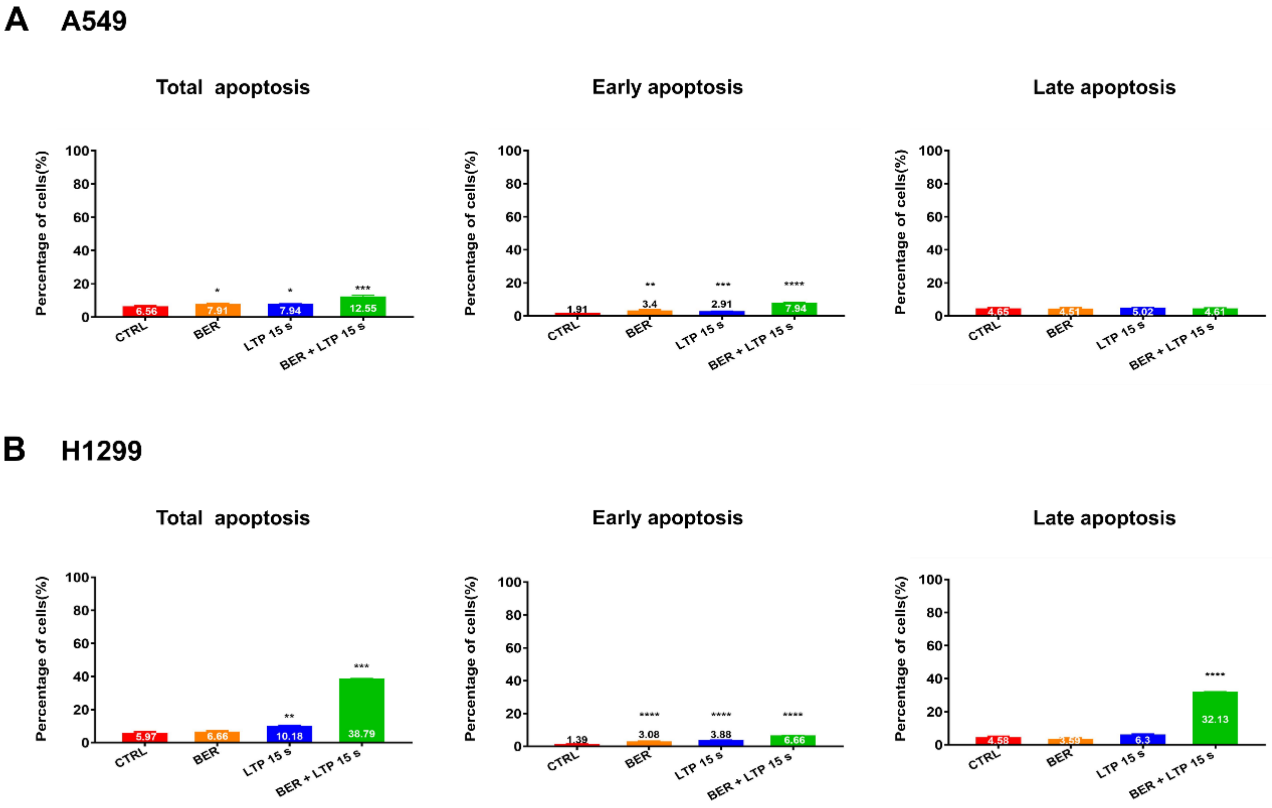

Supplementary Figure S2. Effects of berberine and/or LTP on cell apoptosis (Flow cytometry). (A) BER and/or LTP treatment affected the cell apoptosis in A549 cells. (B) BER and/or LTP treatment affected the cell apoptosis in H1299 cells. Data were presented as the Mean ± SEM of three independent experiments. Statistical significance was calculated using T-test to compare each treatment group to the corresponding vehicle control; significance is indicated by asterisks: *p < 0.05, **p < 0.01, ***p < 0.001, ****p < 0.0001.

Supplementary Table S1. Total RNA purity and integrity determination results[a]

Sample name	Concentration (ng/ µL)	Total (µg)	Result description
A_0_1	570.6	45.65	A
A_0_2	820.5	65.64	A
A_0_3	851	68.08	A
A_0_B1	803.5	32.14	A
A_0_B2	919.8	36.79	A
A_0_B3	571.1	45.69	A
A_30_1	825.9	66.07	A
A_30_2	489	78.24	A
A_30_3	764.6	91.75	A
A_30_B1	672.7	40.36	A
A_30_B2	336.2	20.17	A
A_30_B3	464.4	13.93	A
H_0_1	669.7	107.15	A
H_0_2	402	64.32	A
H_0_3	564.2	90.27	A
H_0_B1	515.6	82.5	A
H_0_B2	455.7	72.91	A
H_0_B3	532	85.12	A
H_15_1	730	146	A
H_15_2	720	144	A
H_15_3	408.5	81.7	A
H_15_B1	437.1	69.94	A
H_15_B2	410.9	65.74	A
H_15_B3	351	56.16	A

[a] A: A549 cells.

H: H1299 cells.

B: berberine treatent.

0: LTP expose for 0 s.

15: LTP expose for 15 s.

30: LTP expose for 30 s.

Supplementary Table S2. Filtered reads quality statistics[a]

Sample	Total Raw Reads (Mb)	Total Clean Reads (Mb)	Total Clean Bases (Gb)	Clean Reads Q20 (%)	Clean Reads Q30 (%)	Clean Reads Ratio (%)
A_0_1	45.57	44.15	6.62	96.88	92.06	96.87
A_0_2	45.57	43.99	6.6	96.9	92.12	96.53
A_0_3	47.33	44.78	6.72	97.02	92.41	94.62
A_0_B1	47.33	44.72	6.71	96.91	92.14	94.48
A_0_B2	47.33	45.39	6.81	97.08	92.54	95.91
A_0_B3	47.33	45.02	6.75	96.95	92.25	95.14
A_30_1	47.33	45.14	6.77	97.33	93.12	95.38
A_30_2	47.33	45.01	6.75	97.3	93.02	95.1
A_30_3	47.33	45.1	6.77	97.22	92.83	95.3
A_30_B1	47.33	45.21	6.78	97.34	93.13	95.53
A_30_B2	47.33	44.86	6.73	97.4	93.26	94.78
A_30_B3	47.33	44.78	6.72	97.36	93.2	94.62
H_0_1	45.57	43.94	6.59	97.21	92.66	96.42
H_0_2	45.57	43.95	6.59	97.26	92.77	96.44
H_0_3	47.33	45.24	6.79	97.37	93.03	95.59
H_0_B1	45.57	43.84	6.58	97.31	92.91	96.2
H_0_B2	47.33	45.4	6.81	97.34	92.98	95.94
H_0_B3	47.33	44.99	6.75	96.98	92.35	95.06
H_15_1	47.33	44.54	6.68	97.02	92.41	94.12
H_15_2	47.33	44.74	6.71	96.94	92.24	94.53
H_15_3	47.33	45.47	6.82	96.83	91.98	96.08
H_15_B1	45.57	44.18	6.63	96.97	92.25	96.93
H_15_B2	45.57	43.92	6.59	96.97	92.3	96.37
H_15_B3	47.33	45.38	6.81	97	92.39	95.89

[a] Total Raw Reads (Mb): reads before filtering. Total Clean Reads (Mb): reads after filtering. Total Clean Bases (Gb): total number of base after filtering; Clean Reads Q20 (%): the proportion of alkali base with mass value greater than 20 in filtered reads to total alkali base. Clean Reads Q30 (%): the proportion of alkali base with mass value greater than 30 in filtered reads to total alkali base. Clean Reads Ratio (%): filtered reads ratio.

Supplementary Table S3. Statistical table of reference genome comparison results[a]

Sample	Total Clean Reads (Mb)	Total Mapping(%)	Uniquely Mapping(%)
A_0_1	44.15	87.86	82.66
A_0_2	43.99	87.65	82.49
A_0_3	44.78	87.17	81.93
A_0_B1	44.72	87.24	82.04
A_0_B2	45.39	87.46	82.11
A_0_B3	45.02	87.27	82.04
A_30_1	45.14	86.87	81.59
A_30_2	45.01	87.2	81.84
A_30_3	45.1	86.71	81.39
A_30_B1	45.21	87.52	82.08
A_30_B2	44.86	87.14	81.65
A_30_B3	44.78	88.11	82.96
H_0_1	43.94	90.07	84.08
H_0_2	43.95	89.8	83.84
H_0_3	45.24	89.85	83.76
H_0_B1	43.84	90.97	84.84
H_0_B2	45.4	90.75	84.6
H_0_B3	44.99	89.55	83.68
H_15_1	44.54	88.64	82.63
H_15_2	44.74	88.67	82.78
H_15_3	45.47	88.67	82.73
H_15_B1	44.18	89.57	83.48
H_15_B2	43.92	89.57	83.47
H_15_B3	45.38	89.45	83.29

[a] Total Clean Reads: reads after filtering. Total Mapping: Clean Reads ratio of reference genome on comparison. Uniquely Mapping: The proportion of Clean Reads at a certain position of the reference genome in the unique comparison.

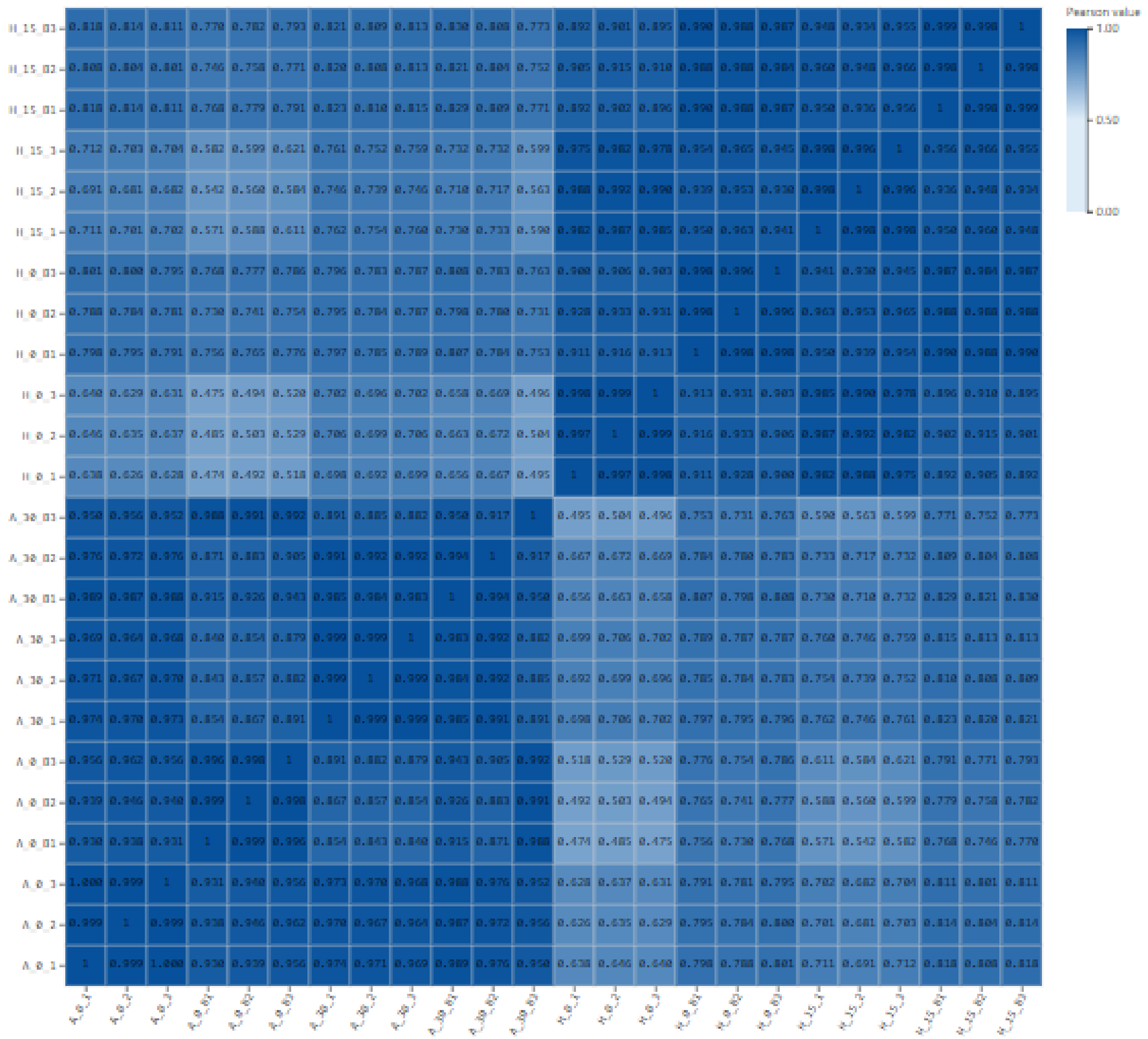

Supplementary Figure S3. Correlation between A549 and H1299 cell samples. The X-axis and Y-axis represent each sample, and the color represents the correlation coefficient. The darker the color is, the higher the correlation is. The lighter the color is, the lower the correlation is.

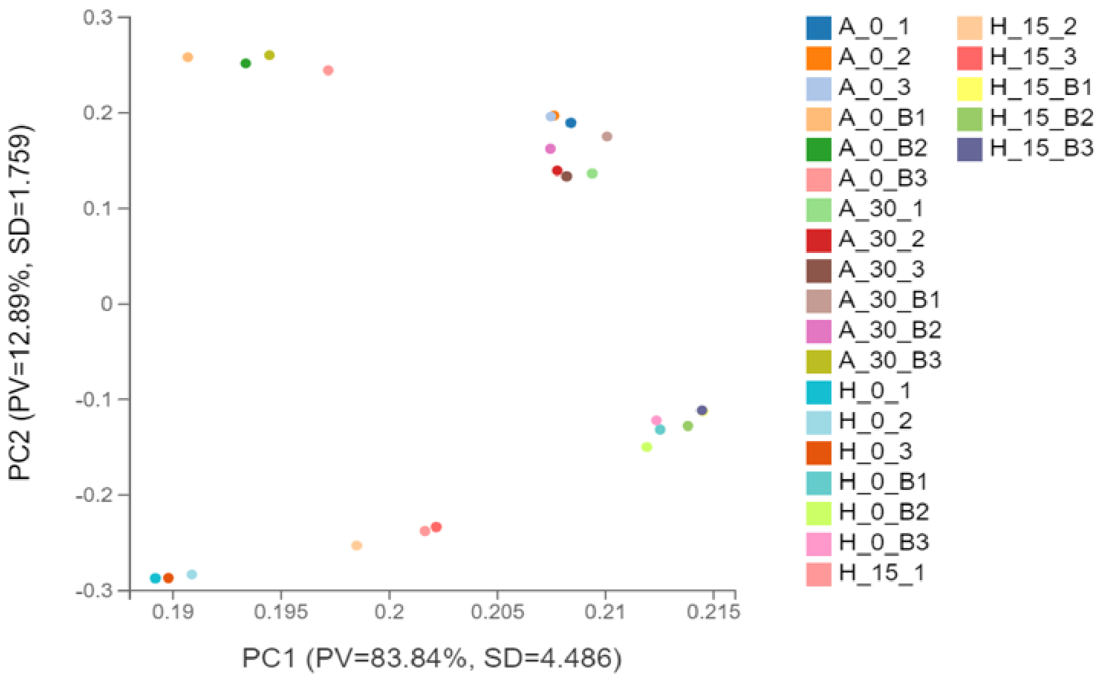

Supplementary Figure S4. PCA analysis results. The X-axis and Y-axis represent the new data set corresponding to the principal component of the sample expression volume after dimension reduction, which is used to represent the gap between samples; The values in the brackets of the coordinate axis labels represent the percentage of the population variance explained by the corresponding principal components. The point represents each sample, and the same color represents the same sample group. PV stands for Proposal of variance, and SD stands for standard deviation.

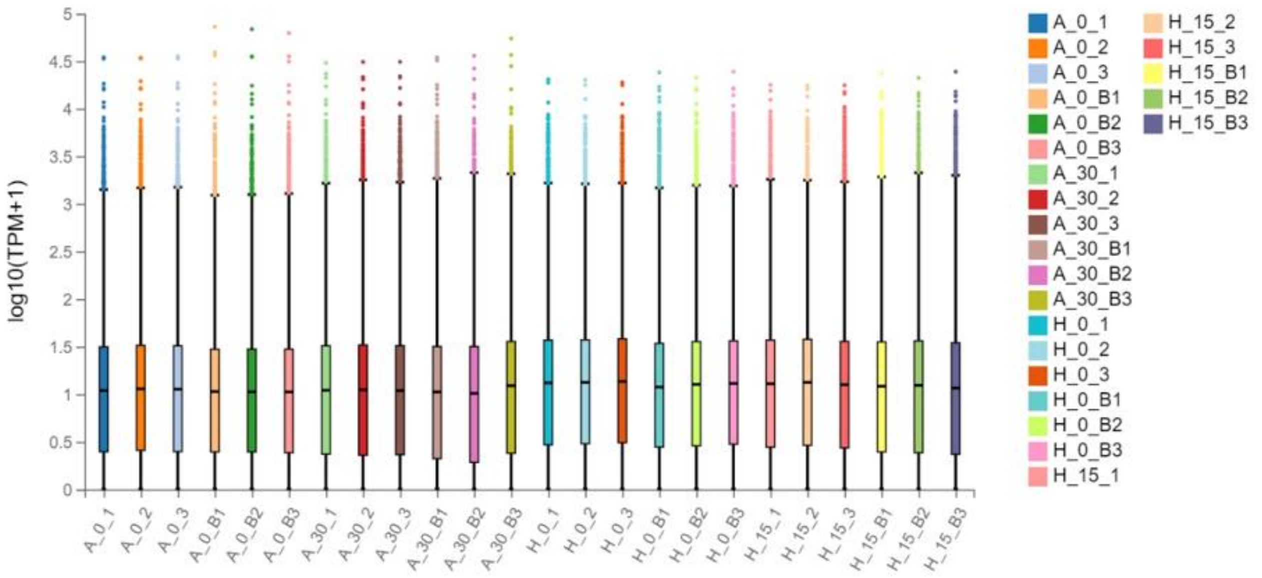

Supplementary Figure S5. Distribution of gene expression levels of each sample. The X axis is the name of the sample, the Y axis is log10 (TPM+1), and the boxplot of each area corresponds to five statistics (from top to bottom, they are respectively the upper limit, upper quartile, median, lower quartile, and lower limit, where the upper limit and lower limit do not take into account abnormal values)

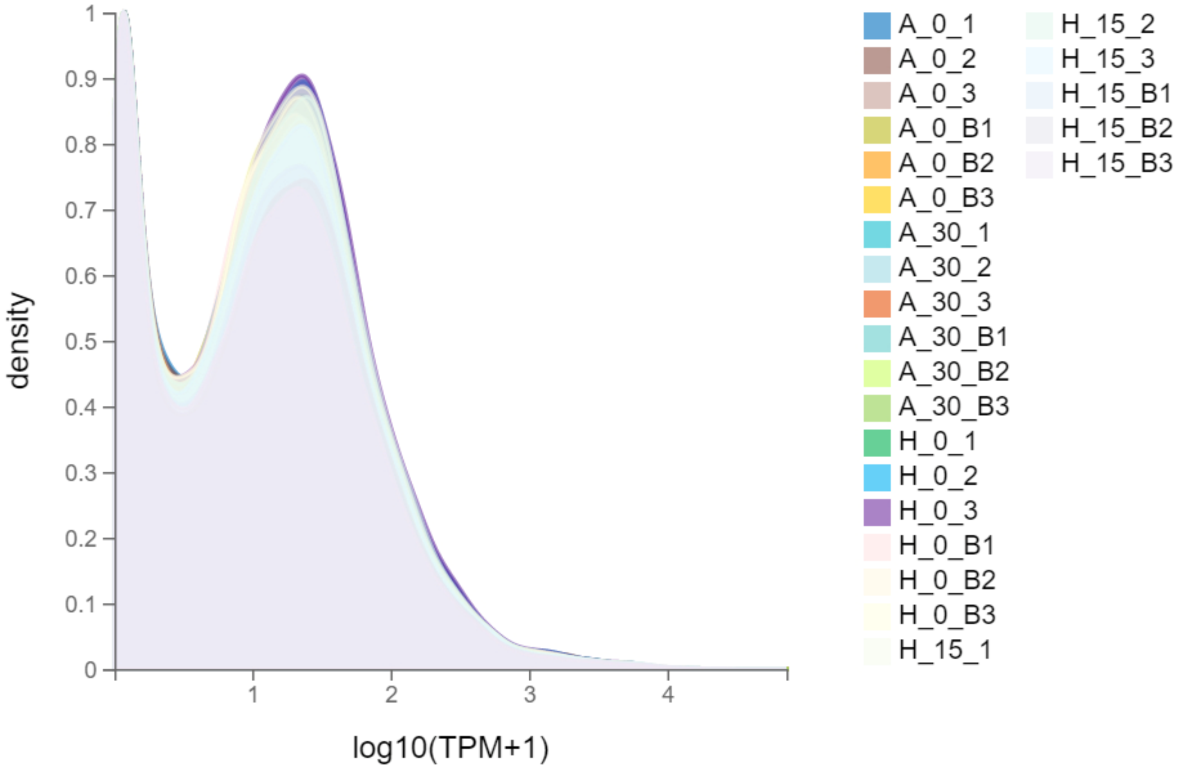

Supplementary Figure S6. Density diagram of expression volume of each sample. The X axis is log10 (TPM+1), and the Y axis is the density of genes, that is, the ratio of the number of genes under this expression amount to the total number of genes expressed.

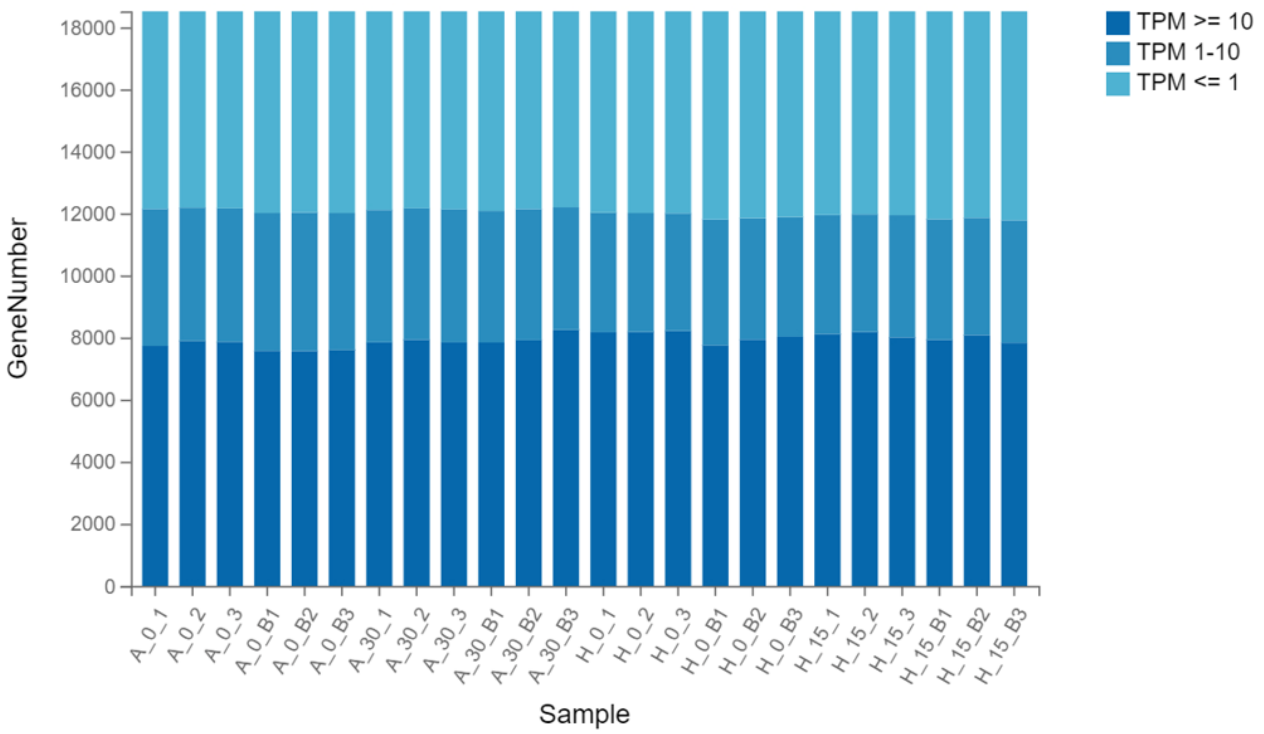

Supplementary Figure S7. Stacking map of gene expression of each sample. The XX axis represents the name of the sample, the Y axis represents the number of genes, and the color depth represents different levels of expression: TPM $\leq$ 1 is a very low expression level gene, TPM between 1-10 is a low expression level gene, TPM $\geq$ 10 is a medium high expression level gene.

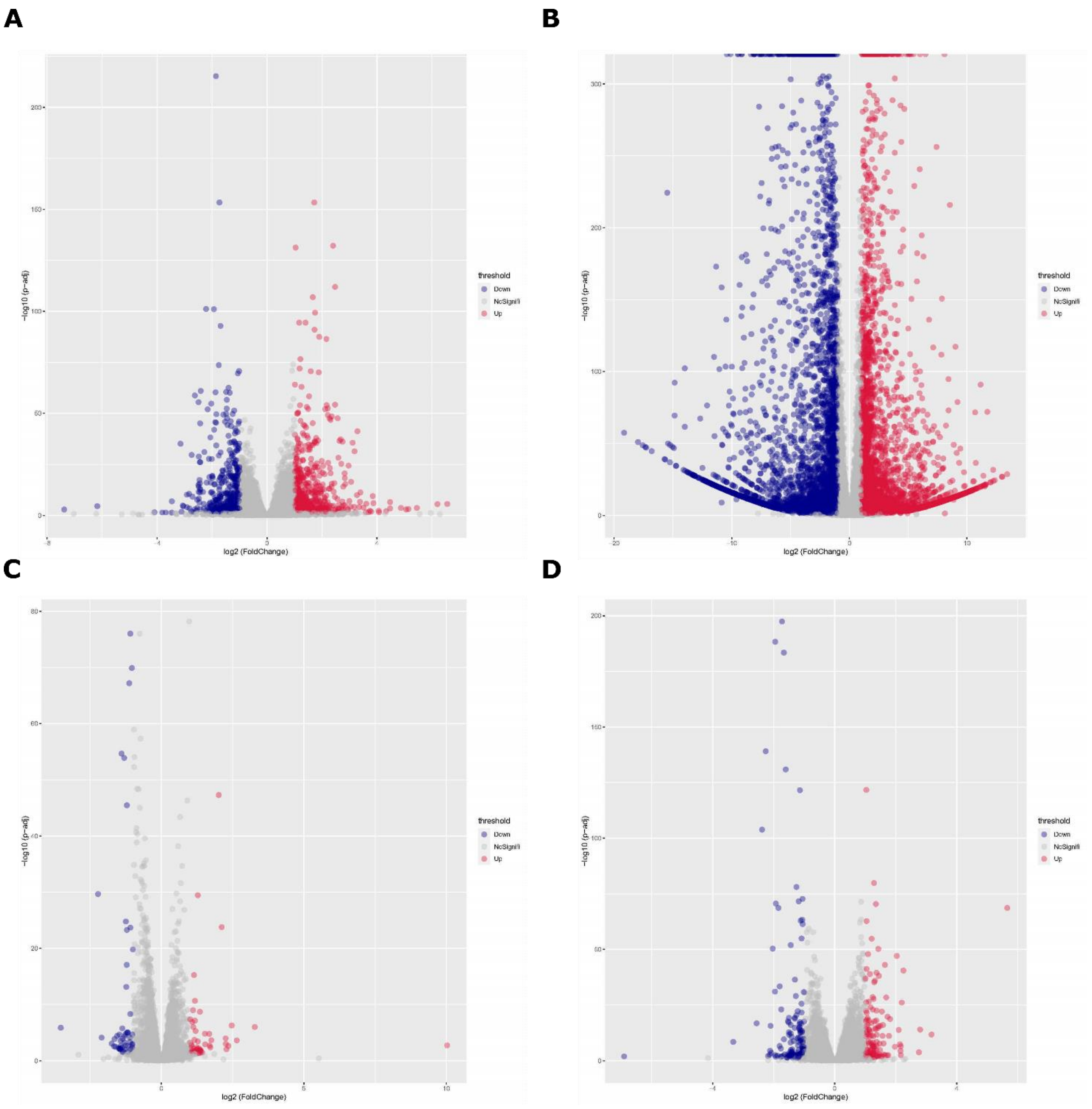

Supplementary Figure S8. Volcanic map showing gene differences between groups. (A) Intersection of high and low expression genes in the LTP treatment group and control group of A549 cells. (B) Intersection of high and low expression genes in the control group of A549 cells and H1299 cells. (C) Intersection of high and low expression genes in the LTP treatment group and control group of H1299 cells. (D) Intersection of high and low expression genes between the LTP treatment group and the BER combined with LTP group on H1299 cells.

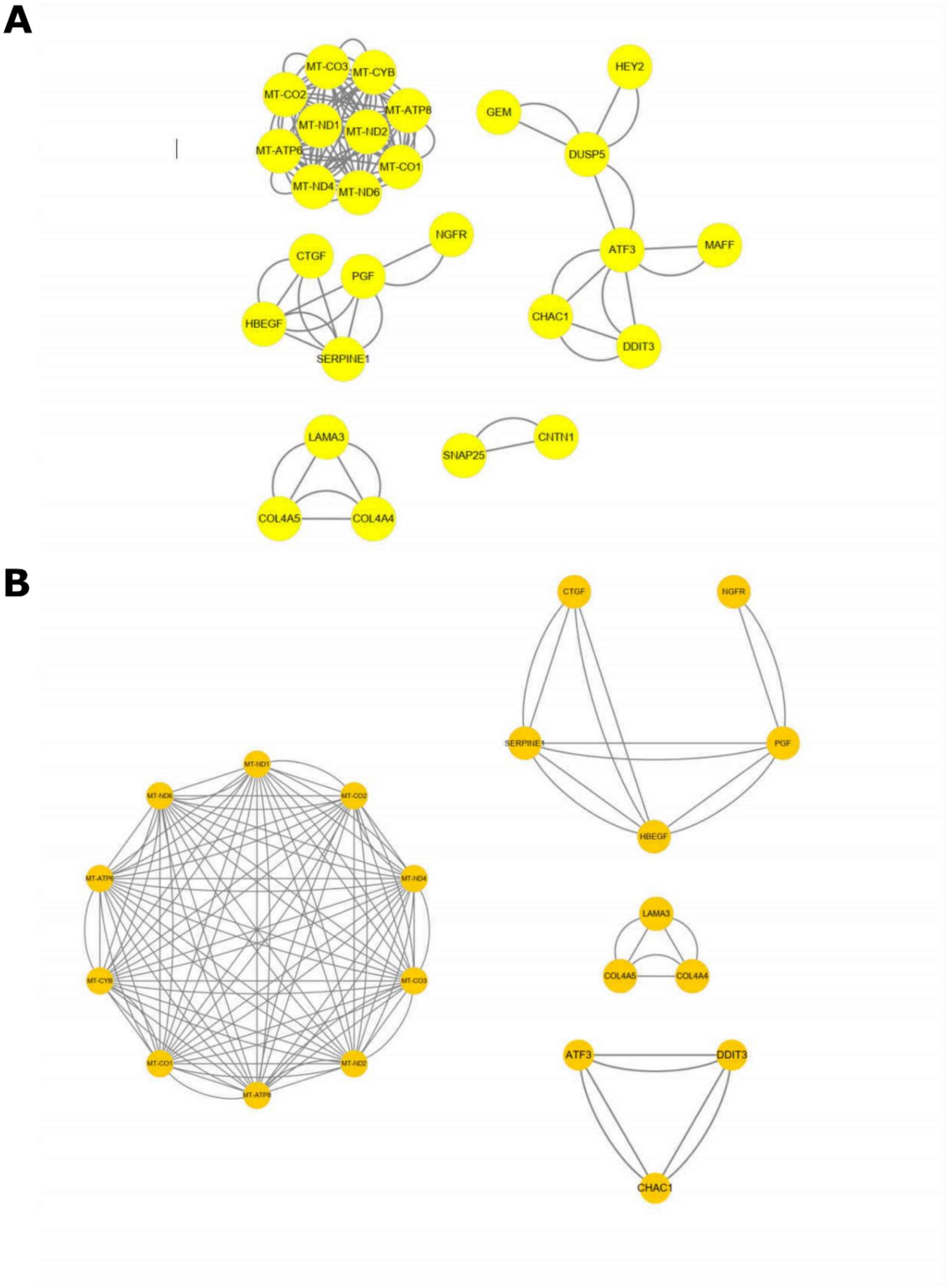

Supplementary Figure S9. Protein interaction network diagram. (A) The general diagram. (B) The subnet analysis done by the MCODE plug-in of the cycloscape software. The genes of each subnet can be regarded as hub genes.
